# Supplementary material for: Dietary (Poly)phenols in Traumatic Brain Injury
Source: Int J Mol Sci. 2023 May 17;24(10):8908. doi: 10.3390/ijms24108908 (PMC10219504; doi:10.3390/ijms24108908)
Supplement: Supplementary file 1 [file ijms-24-08908-s001.zip › ijms-2373965-supplementary.pdf]

Table S1. Flavanols effects on TBI models after oral administration. TBI: traumatic brain injury; CCI: controlled cortical impact; WT: Wild-type; BBB: blood-brain barrier; ZO-1: zonula occludens 1; IL-1β: interleukin 1 beta; IL-6: interleukin 6; iNOS: inducible nitric oxide synthase; Nrf2: nuclear factor-erythroid factor 2–related factor; HO-1: heme oxygenase 1; ROS: reactive oxygen species; MMP9: matrix metalloproteinase; Keap1: Kelch-like ECH-associated protein 1; SOD1: superoxide dismutase; Iba-1: ionized calcium-binding adaptor molecule 1; GFAP: glial fibrillary acidic protein; TNF-α : tumor necrosis factor alpha; IKK: inhibitor of nuclear factor-κB (IκB) kinase; NF-κB: nuclear factor kappa B; MDA: malondialdehyde; Hpp: hippocampus; CAT: catalase; GSH: glutathione; AMPK: 5' AMP-activated protein kinase; 4-HNE; 8-OhdG: 8-hydroxy-2′-deoxyguanosine; d: day; h: hour.

Flavanols	TBI model	Species	Route/ concentration	Regimen	Time of the brain collection	Main results	Reference
Catechin 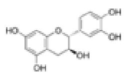	CCI	Sprague–Dawley rats	1, 5, 10, 20 or 30 mg /kg body weight	Once per day after TBI	24 h after TBI	↑neurological outcomes (motor and cognitive)<br>↓brain tissue damage<br>↓ brain edema<br>↑ BBB integrity<br>↑ Ocludin and ZO-1 mRNA and protein levels<br>↓ IL-1β, IL-6, iNOS mRNA expression<br>↑ arginase-1 mRNA expression	[83]
(−)-epicatechin 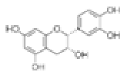	CCI	C57BL/6 WT and Nrf2 KO mice	Short-term observation (3 days): 5, 15, or 45 mg/kg	3 h after TBI and once daily for 3 days	1 and 3 days after TBI	↑ neurologic function (3 and 28d)<br>↑ cognitive performance (3 and 28d)<br>Improved depression-like behaviors (3 and 28d)<br>↓ brain edema (3d)<br>↓ lesion volume (3 and 28d)<br>↓ white matter injury (28d)<br>↓ neuronal cell death (3d)<br>↓ OH-1 expression (3d)<br>↓ferric iron deposition (3d)<br>↓neutrophil infiltration (1d)<br>↓ROS levels (3d)<br>↓activity of MMP9 (1d)<br>↓Keap 1 expression(3d)<br>↑Nrf2 nuclear accumulation (3d)<br>↑ SOD1 expression (3d)<br>↑quinone 1 expression (3d)	[84]
			Long-term observation (7 days): 15 mg/kg	3 h after TBI and once daily 7 days	28 days after TBI		
(-)-Epigallocatechin-3-gallate (EGCG) 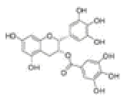	CCI	C57BL/6 mice (, AMPKα1flox/ flox mice, and RosaCre-ER mice	0.2% (w/v) dissolved in drinking water	Immediately after TBI and accessible till 7 days	7 days after TBI	↑ neurological score (including motor performance and spatial learning and memory)<br>↓ Iba-1 and GFAP mRNA levels in peri-injured area<br>↓ number of activated microglial and astroglial cells in peri-injured area<br>↓ TNF-α , IL-1β, IL-6 serum and brain protein levels in peri-injured area<br>↓ phosphorylation of IKKα/β, IκBα and nuclear translocation	[85]

					of NF-κB p65 in peri-injured area<br>↓ O_2, H_2O_2 and MDA levels in the Hpp<br>↑ SOD, CAT and GSH levels in the Hpp<br>↑ AMPK phosphorylation	
CCI	Wistar rats	0.1% (w/v) dissolved in drinking water	Ad libitum access 4 weeks before TBI	1, 3 and 7 days after TBI	↑ Morris water maze performance (3,4 and 5d)<br>↓ number of apoptotic cells around damaged region (ssDNA-labelling) (1, 3 and 7d)<br>↓ DNA damage (8-OHdG labelling) (1, 3 and 7d)<br>↓ lipid peroxidation in neurons (4-HNE labelling) (1 and 3d)<br>↓ MDA levels (1 and 3d)<br>↑ NeuN positive cells around the injury (7d)<br>↓ glial scar (GFAP labelling) (7d)	[71]
			Ad libitum access to EGCG 4 weeks before TBI	1, 3 and 7 days after TBI	↑ number of neural stem cells around the damaged area (nestin-positive cells) (1,3 and 7d)<br>↓ MDA levels (1 and 3d)<br>↓ lipid peroxidation and oxidative damage (4-HNE labelling) (1 and 3d)<br>↓ DNA damage (8-OHdG labelling) (1,3 and 7d)<br>↓ neuronal cell apoptosis (ssDNA-labelling) (1,3 and 7d)	[72]
			Tree protocols: were used: i) 4 weeks before TBI (pre-treatment)<br>ii) 4 weeks before TBI and 1 week after TBI (continuous treatment)<br>iii) only 1 week after TBI (post-treatment)	1, 3 and 7 days after TBI	↑ Morris water maze performance (only in continuous treatment and post-treatment groups) (4 and 5d)<br>↓ MDA levels (1 and 3d)<br>↓ lipid peroxidation and oxidative damage (4-HNE labelling) (1 and 3d)<br>↓ DNA damage (8-OHdG labelling) (1,3 and 7d)<br>↓ neuronal cell apoptosis (ssDNA-labelling) (1,3 and 7d)<br>↑ number of Bcl-2 positive cells around the damaged area (1 and 3d)<br>↑ NeuN positive cells around the injury (7d)<br>↓ glial scar (GFAP labelling) (7d)	[73]

Table S2. Flavanones effects on TBI models after oral and intraperitoneal administration. TBI: traumatic brain injury; IL-1β: interleukin 1 beta; TNF-α : tumor necrosis factor alpha; MDA: malondialdehyde; Hpp: hippocampus; BDNF: brain-derived neurotrophic factor; IL-6: interleukin 6; iNOS: inducible nitric oxide synthase; Nrf2: nuclear factor-erythroid factor 2–related factor; ER: endoplasmic reticulum; GRP78: Glucose-regulated protein; ATF4: activating transcription factor 4; p-eIF2α: phosphorylated eukaryotic translation initiation factor 2α; d: day; h: hour; n.s.: not specified.

Flavanones	TBI model	Species	Route/ concentration	Regimen	Time of the brain collection	Main results	Reference
Hesperidin 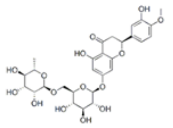	Weight drop	NMRI mice	Oral gavage/ 50 mg/kg	Once daily starting 10 days after TBI and lasting for 14 days	Brain collection time (not specified)	↓ TBI-induced depression-related symptoms<br>↓ IL-1β, TNF-α protein levels in Hpp<br>↓ MDA protein levels in Hpp<br>↑ BDNF protein levels in Hpp	[88]
Naringenin 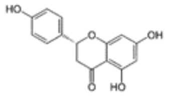	Weight drop	ICR mice	IP/ 50 and 100 mg/kg	Immediately after TBI, and then daily for 3 or 7 consecutive days	6h,1, 3,5 and 7 days after TBI	↓ neurological dysfunction (3,5 and 7d)<br>↓ brain edema (3d)<br>↓ plasmalemma permeability (n.s.)<br>↓ neuronal cell loss (n.s.)<br>↑ Bcl-2 protein levels (n.s.)<br>↓ Bax and caspase-3 protein levels (n.s.)<br>↓ ER stress pathway (GRP78, ATF4, and CHOP) (n.s.)<br>↑ p-eIF2α<br>↓ MDA protein level (6h, 1,3 and 5d)<br>↑ GPx protein level (6h, 1,3 and 5d)	[87]

Table S3. Flavonols effects on TBI models after oral and intraperitoneal administration. TBI: traumatic brain injury; FPI: fluid percussion injury; IP: intraperitoneal injection; HPA: hypothalamic–pituitary–adrenal; TNF-α : tumor necrosis factor alpha; iNOS: inducible nitric oxide synthase; IL-1β: interleukin 1 beta; IL-6: interleukin 6; IL-10: interleukin 10; MDA: malondialdehyde; CAT: catalase; GPx: glutathione peroxidase; SOD: superoxide dismutase; Nrf2: nuclear factor-erythroid factor 2–related factor; HO-1: heme oxygenase 1; TBARS: thiobarbituric acid reactive substance; TUNEL: terminal deoxynucleotidyl transferase (TdT) dUTP Nick-End labeling; PGC-1α: peroxisome proliferator-activated receptor-gamma coactivator-1α; ATP: adenosine triphosphate MPO: Myeloperoxidase; GSH: glutathione; ARE: antioxidant responsive element; BBB: blood-brain barrier; AChE: acetylcholinesterase; Hpp: hippocampus; d: day; h: hour.

Flavonols	TBI model	Species	Route/ concentration	Regimen	Time of the brain collection	Main results	Reference
Quercetin 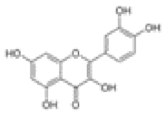	Weight drop	Naval Medical Research Institute mice	Oral gavage/ 50mg/kg	Once daily starting 10 days after TBI and lasting next 14 days	24 days after TBI	↓ anxiety-like behaviours<br>↓ dysregulation of the HPA axis<br>↓ adrenocorticotropic hormone and corticosterone	[102]

Weight drop	Sprague Dawley rats	IP / 50 mg/kg	30 min, 12 and 24 h following the TBI	1, 3 and 5 days after TBI	↓ brain edema (1,3 and 5d)<br>↑motor function (1,3 and 5d)<br>↓ neuronal apoptosis (1d)<br>↓ caspase-3 protein levels (1d)<br>↑ Akt serine/threonine protein kinase phosphorylation (1d)<br>↓ ERK ½ phosphorylation (1d)	[99]
Weight drop	Sprague Dawley rats	IP / 50 mg/kg	30 min, 12 and 24 h following the TBI	48h after TBI	↓ neurological impairment<br>↑ cognitive function<br>↓ neuronal autophagy<br>↑ p-Akt protein levels<br>↑ Bcl-2 protein levels	[100]
Weight-drop	Sprague Dawley rats	IP / 5, 20 or 50 mg/kg	0.5, 12 and 24 h post TBI	3 days after TBI	↓ brain edema<br>↓ microgliosis<br>↓ TNF-α , iNOS, IL-1β and IL-6 mRNA and protein levels in serum<br>↓ TNF-α , iNOS, IL-1β and IL-6 mRNA levels in the brain<br>Restored MDA, CAT, GPx and SOD levels in the brain<br>↑ nuclear and total Nrf2 levels in the brain<br>↑ HO-1 protein levels	[90]
Weight-drop	Sprague–Dawley rats	IP/30 mg/kg	0, 24, 48, and 72 h after TBI	7 and 28 days after TBI	↓ cognitive deficits (25-28d)<br>↓ number of ED-1-positive cells (7 and 28d)<br>↓ number of TUNEL-positive cells (7d)<br>↓ Bax and cleaved-caspase-3 protein levels (7d)<br>↓ TNF-α , IL-1b and IL-6 protein levels (7d)<br>↑ IL-10 protein levels (7d)<br>↑ GPx, SOD, and CAT activity (7d)<br>↓ lipid peroxidation (TBARS levels) (7d)	[91]
Weight-drop	ICR mice	IP/ 20, 50 and 100 mg/kg	30 min after TBI	24h after TBI	↓ brain edema<br>↓ neuronal apoptosis<br>↓ Bax and cleaved-caspase-3 protein levels<br>↓ mitochondrial lesions	[98]

						↑ PGC-1α protein translocation to nucleus<br><br>Restored cytochrome c, MDA and SOD levels in mitochondria	
	Weight-drop	ICR mice	IP/ 50 mg/kg	30 min after TBI	24h after TBI	↑ Nrf2 total protein levels<br>↑ Nrf2 translocation to nucleus<br>Restored MDA levels, SOD activity, mitochondrial membrane potential and intracellular ATP content<br>Restored levels of cytochrome c and Bax in cytosol and mitochondria	[94]
	FPI	Sprague-Dawley rats	IP/ 25 μmol/kg	1 h after injury with continued treatment at 12-h intervals	1 and 3 days after TBI	↑ action potential from corpus callosum (1 and 3d)<br>↓ Myeloperoxidase activity (1d)<br>↑ GSH levels protein levels (1d)	[95]
Fisetin<br>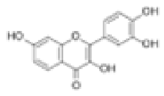	Weight-drop	ICR mice	IP / 25, 50 and 75mg/kg<br><br>(50mg/kg showed best effects)	30 min after TBI	1, 3 and 7 days after TBI	↑ neurological function (1 and 3d)<br>↓ cerebral edema (1d)<br>↓ brain lesion volume (7d)<br>↑ Nrf2 nuclear levels (1d)<br>↑ Nrf2 binding to ARE (1d)<br>↑ BBB integrity (1d)<br>↓ neuroapoptosis (1d)<br>↓cleaved Caspase-3 protein level (1d)<br>↑ Bcl-2 protein level (1d)<br>↓ Bax protein level (1d)<br>Restored MDA and GPx levels (1d)	[97]
Rutin<br>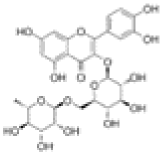	Weight drop	ICR mice	IP/ 30, 50 and 80 mg/kg<br><br>(50mg/kg showed best effects)	30 min after TBI	24h after TBI	↑ neurological function<br>↓ brain edema<br>↓neuronal apoptosis<br>↓ cleaved-caspase-3 protein levels<br>↓ release of cytochrome c to cytoplasm<br>↓ translocation of Bax to mitochondria<br>Restored levels of MDA and SOD	[96]

	Weight drop	Wistar rats	Oral gavage/ 50mg/kg	Once a day for 3 days, starting right after the TBI	4 days after TBI	↓ brain edema<br>↓ NF-κB and TNF-α protein levels<br>↓ neuronal degeneration<br>↓poly-morpho-nuclear-leucocyte accumulation<br>Restored MDA and rGSH levels	[92]
	Weight drop	Wistar rats	Oral gavage/ 20, 40, and 80 mg/kg	Once daily starting 14 days after TBI and lasting next 14 days	28 days after TBI	↑ spatial memory<br>↓ AChE levels<br>↓ caspase-3 protein levels<br>↓ TNF-α protein levels	[93]
Kaempferol<br>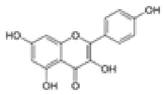	FPI	Sprague–Dawley rats	IP / 1 mg/kg	1, 24, and 48 h after TBI	no brain harvesting	↑ neural connectivity in somatosensory cortex and Hpp<br>↑ neurovascular coupling<br>↑ fractional anisotropy (FA)<br>↑ apparent diffusion coefficient<br>↑ corpus callosal fractional anisotropy<br>↑ parenchymal microstructure	[103]
	FPI	Sprague-Dawley rats	IP / 1 mg/kg	1, 24 and 48 h after TBI	72h after TBI	↑ sensorimotor behavior<br>Altered metabolome of the brain	[101]

Table S4. Flavones effects on TBI models after oral and intraperitoneal administration. TBI: traumatic brain injury; CCI: controlled cortical impact; WT: Wild type; IP: intraperitoneal injection; SOD: superoxide dismutase; CAT: catalase; GPx: glutathione peroxidase; GSH: glutathione; MDA: malondialdehyde; IHC: immunohistochemistry; VCS: Veterinary Coma Scale; TNF-α : tumor necrosis factor alpha; IL-1β: interleukin 1 beta; Aβ: amyloid β; GSK-3: Glycogen synthase kinase-3; NF-κB: nuclear factor kappa B; BBB: blood-brain barrier; Nrf2: nuclear factor-erythroid factor 2–related factor; HO-1: heme oxygenase 1; NQO1: NAD(P)H:quinone oxidoreductase 1; d: day; h: hour.

Flavones	TBI model	Species	Route/ concentration	Regimen	Time of the brain collection	Main results	Reference
Chrysin<br>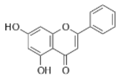	Weight-drop	Wistar rats	Oral gavage / 25, 50 or 100 mg/kg<br>(100mg/kg showed best effects)	Once a day for 3 or 14 days, starting right after	3, 14 and 28 days after TBI	↑ motor coordination (3-14d)<br>↑ learning and memory (3-14d)<br>↑ SOD, CAT, GPx, and GSH protein levels (3 and 14d)<br>↓ MDA content (3 and 14d)<br>↓neuroapoptosis (28d)<br>↑Bcl-2 protein expression (IHC) (28d)<br>↓Bax protein expression (IHC) (28d)	[108]

						No improvement in the VCS	
Luteolin 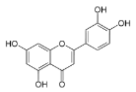	Gas-CCI	Tg2576 mice and litter-mate controls (WT)<br><br>Sex is not specified!	IP / 20 mg/kg	Daily, 15 consecutive days prior to TBI	3 and 14 days after TBI	↓ TNF-α and IL-1β protein levels (3d)<br>↓ Aβ40 and Aβ42 protein levels (3d)<br>↓ GSK-3 activation (3d)<br>↓ phospho-tau levels (3d)	[76]
	Weight-drop	CD1 mice	IP/ 30mg/kg	Single injection 30 min after the TBI	24 h after TBI	↓ brain edema<br>↓ IL-1b and TNF-a mRNA and protein levels<br>↓ NF-kB p65 nuclear accumulation<br>↓ neuronal degeneration<br>↑ BBB integrity<br>↑ autophagy	[106]
	Weight-drop	ICR mice	IP / 10, 30, and 50 mg/kg<br><br>(30mg/kg showed best effects)	Single injection 30 min after the TBI	24 h after TBI	↓ neurological deficits<br>↓ brain edema<br>Restored MDA levels and GPx activity<br>↑ Nrf2 nuclear content<br>HO1 and NQO1 mRNA and protein levels<br>↓ neuronal apoptosis	[107]

Table S5. Genistein effects on TBI model after intraperitoneal administration. TBI: traumatic brain injury; IP: intraperitoneal injection; BBB: blood-brain barrier; h: hour.

Isoflavones	TBI model	Species	Route/ concentration	Regimen	Time of the brain collection	Main results	Reference
Genistein 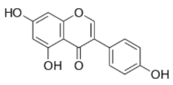	Weight drop	Wistar rats	IP/ 15 mg/kg	30 min and again 24 h after TBI	48h after TBI	↓ brain edema<br>↓ BBB permeability<br>↓ intracranial pressure<br>↑ motor performance	[109]

Table S6. Curcumin effects on TBI models after oral and intraperitoneal administration. TBI: traumatic brain injury; FPI: fluid percussion injury; CCI: controlled cortical impact; IP: intraperitoneal injection; pAMPK: phosphorylated 5' adenosine monophosphate-activated protein kinase; uMtCK: ubiquitous mitochondrial creatine kinase; COX-II: cytochrome c oxidase II; UCP-2: uncoupling protein 2; BDNF: brain-derived neurotrophic factor; CREB: cAMP-response element binding protein; MDA: malondialdehyde; IL-1β: interleukin 1 beta; IL-6: interleukin 6; TNF-α : tumor necrosis factor alpha; Nrf2: nuclear factor-erythroid factor 2–related factor; HO-1: heme oxygenase 1; NQO1: NAD(P)H:quinone oxidoreductase 1; GCLC: glutamate-cysteine ligase catalytic subunit; GCLM: glutamate-cysteine ligase modifier subunit; Hpp: Hippocampus; IL-18: interleukin 18; p-Trkb: phosphorylated tropomyosin receptor kinase B; PI3K: phosphoinositide 3-kinases; TLR4: Toll-like receptor 4; NF-κB: nuclear factor kappa B; p-IκB-α: phosphorylated nuclear factor of kappa light polypeptide gene enhancer in B-cells inhibitor; GFAP: glial fibrillary acidic protein; AQP4: Aquaporin-4; d: day; h: hour.

Curcuminoids	TBI model	Species	Route/ concentration	Regimen	Time of the brain collection	Main results	Reference

	FPI	Sprague – Dawley rats	Rat chow enriched with 500 ppm of curcumin	Four weeks before, and 1 week after TBI	7 days after TBI	↑pAMPK protein levels<br>↑ uMtCK protein levels<br>↑ COX-II protein levels<br>↑ UCP-2 protein levels<br>↑ SIR-2 protein levels	[77]
	FPI	Sprague – Dawley rats	Rat chow enriched with 500 ppm of curcumin	Four weeks before TBI, and 1 week after TBI	7 days after TBI	↑ cognitive performance<br>↓oxidized protein levels<br>↑ BDNF protein levels<br>↑ synapsin I and p-synapsin I protein levels<br>↑ CREB protein levels	[78]
	Weight drop	Wistar rats	IP / 50 and 100 mg/kg	Daily, 5 days before TBI	14 days after TBI	↓ animal death rate<br>↑ motor and cognitive performance<br>↓ lesion volume<br>↓ MDA levels	[79]
Curcumin<br>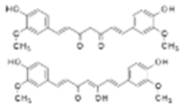	Weight drop	C57BL/6 mice	IP / 50 mg/kg	Single injection 15 min after TBI	24h after TBI	↓ brain edema<br>↓ ipsilateral cortex injury<br>↓ microglial activation<br>↓ IL-1β, IL-6, TNF-α mRNA levels<br>↑ Nrf2 protein expression and nuclear translocation<br>↑ HO-1, NQO1, GCLM, GCLC mRNA levels<br>↓ neuroapoptosis<br>↓ cleaved caspase-3 protein level<br>↑ Bcl-2 protein level	[111]
	CCI	Sprague/Dawley rats	IP / 30 mg/kg	Daily, 28 days after TBI	7, 14, 21, 28 and 40 days after TBI	↑ spatial memory (40d)<br>↑neurogenesis in Hpp (35d)<br>↓ IL-1β, IL-6, IL-18, TNF-α protein levels (28d)<br>↓microglial and astrocytic activation (28d)<br>↑BDNF, p-Trkb, p-PI3K and p-Akt (7, 14, 21 and 28d)	[113]
	Weight drop	C57BL/6 and transgenic TLR4−/− mice	IP / 100 mg/kg	Single injection, 15 min after TBI	6, 12, 24, 48 and 72h after TBI	↑ neurological score (24h)<br>↓ brain edema (24h)<br>↓ activation of TLR4- positive microglia and astrocytes (24h)<br>↓ IL-1β, IL-6, TNF-α protein levels (24h)<br>↓ TLR4 protein level (24h)<br>↓ MyD88 protein level (24h)<br>↓ NFκB p65 and p-IκB-α protein levels (24h)<br>↓neurodegeneration and neuronal apoptosis (24h)	[112]
	CCI	CD-1 mice (8-10 weeks old)	IP / 75, 150, 300 mg/kg	Single injection, either 15 minutes prior to TBI or post-	Multiple time points from 1h to 21 days after TBI	↑ neurological function (1, 3, 7, 14, 21 and 28d)<br>↓brain edema (24h)	[80]

			(Pre-treatment with 150mg/kg showed best effects)	treated (0.5 or 1h post-TBI)		↓ IL-1β mRNA expression (1, 6 and 12h)<br>↓ GFAP protein expression (24h)<br>↓ AQP4 protein levels (1 and 2d)<br>↓ reduction of NF-κB p65 protein expression in astrocytes (24h)	

Table S7. Resveratrol effects on TBI models after oral and intraperitoneal administration. TBI: traumatic brain injury; CCI: controlled cortical impact; IP: intraperitoneal injection; SC: subcutaneous injection; ROS: reactive oxygen species; Nrf2: nuclear factor-erythroid factor 2–related factor; HO-1: heme oxygenase 1; Hpp: Hippocampus; NLRP3: NLR family pyrin domain containing 3; IL-1β: interleukin 1 beta; IL-18: interleukin 18; SOD: superoxide dismutase; GSH: glutathione; MDA: malondialdehyde; NSE: neuron-specific enolase; IL-12: interleukin 12; GPx: glutathione peroxidase; 8-OhdG: 8-hydroxy-2′-deoxyguanosine; TLR4: Toll-like receptor 4; GSH: glutathione; MDA: malondialdehyde; NO: nitric oxide; XO: xanthine oxidase; IHC: immunohistochemistry; NF-κB: nuclear factor kappa B; TNF-α : tumor necrosis factor alpha; SYN: synuclein; PDS-95: postsynaptic density protein-95; d: day; h: hour.

Stilbenes	TBI model	Species	Route/ concentration	Regimen	Time of the brain collection	Main results	Reference
Resveratrol<br>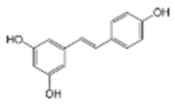	CCI	Sprague-Dawley rats	Oral gavage/0.05 and 0.1mg/kg<br>(0.1 mg/kg showed best effects)	Daily, for 10 consecutive days, starting 7 days after the TBI	not specified	↑ cognitive performance<br>↓ ROS generation<br>↑ Nrf2 protein level<br>↑ HO1 protein level<br>↓ apoptosis<br>↓ cleaved caspase-3 protein level	[119]
	Concussion injury	Wistar rat	IP / 100 mg/kg	Single injection immediately after TBI	17 days after TBI	↑cortex/Hpp dependent memory<br>↓anxiety<br>↓ Hpp neuronal loss	[122]
	Concussion injury	Sprague-Dawley rats	IP / 100 mg/kg	Single injection, 30 min before TBI	24 after TBI	↓ brain edema<br>↓ NLRP3 and caspase-1 mRNA and protein levels<br>↓ IL-1β and IL-18 mRNA and protein levels<br>↑ SOD, GSH protein levels<br>↓ MDA protein levels<br>↓ ROS production<br>↑ SIRT1 protein levels<br>↓ NSE serum levels	[81]
	CCI	Sprague-Dawley rats	IP / 10 and 100 mg/kg<br>(100 mg/kg showed best effects)	Injection 5 min after TBI, and two more injections on post-injury days 1 and 2	21 days after TBI	↑motor performance<br>↑ cognitive performance<br>↓ cortical contusion volume<br>↑neuroprotection in Hpp CA1 and CA3 regions	[120]

CCI	C57Bl/6 mice	SC / 100 mg/kg	Injection 5 minutes and 12 hours after TBI	72h after TBI	↓ microglial activation<br>↓ IL-6 and IL-12 protein levels in Hpp	[116]
Weight drop	Wistar rats	IP / 50 and 100 mg/kg<br><br>(100 mg/kg showed best effects)	Daily, for 7 days after TBI	8 days after TBI	↑ neuroprotection in Hpp<br>↑ GPx and 8-OHdG levels	[118]
Weight drop	Wistar rats	IP / 100 mg/kg	Single injection, immediately after TBI	24h and 14 days after TBI	↓ brain edema (24h)<br>↓ lesion area (14d)<br>↑ GSH protein levels (24h)<br>↓ MDA protein levels (24h)<br>↓ NO and XO protein levels (24h)	[117]
CCI	Sprague-Dawley rats	IP / 100 mg/kg	Bidaily, for 3 days, beginning immediately after TBI	12, 24, 48 and 72h after TBI	↑ spatial cognitive function<br>↑ neurological score (1-5d)<br>↑learning and memory (3-5d)<br>↓ brain edema (12, 24, 48 and 72h)<br>↑ neuroprotection in Hpp (24h)<br>↓ neuronal autophagy LC3/NeuN IHC (24h)<br>↓ neuronal autophagy (LC3I/II, Beclin1) (12,24,48 and 72h)<br>↓ TLR4 neuronal protein levels (TLR4/NeuN) IHC (24h)<br>↓ TLR4 Hpp protein levels (12,24,48 and 72h)<br>↓ NF-kB p65 protein levels (12,24,48 and 72h)<br>↓IL-1β and TNF-α protein levels (12,24,48 and 72h)	[115]
Weight drop	Sprague-Dawley rats	IP / 100 mg/kg	Daily, for 5 days, beginning immediately after TBI	1, 3 and 5 days after TBI	↑ spatial cognitive function (3-5d)<br>↑ neurological score (1-5d)<br>↓ brain edema (1,3 and 5d)<br>↑ synaptic protein levels (SYN, PSD-95) (1,3 and 5d)<br>↓neuronal autophagy (LC3, Beclin1) (1,3 and 5d)	[114]

Table S8. Phenolic acids effects on TBI models after oral and intraperitoneal administration. TBI: traumatic brain injury; CCI: controlled cortical impact; IP: intraperitoneal injection; IL-1β: interleukin 1 beta; IL-6: interleukin 6; BBB: blood-brain barrier; PS: Population spike amplitude; fEPSP: Field excitatory postsynaptic potential; TNF-α : tumor necrosis factor alpha; MDA: malondialdehyde; HDAC2: histone deacetylase 2; AUC: area under curve; GSH: glutathione; SOD: superoxide dismutase; GPx: glutathione peroxidase; F2-IsoPs: F2-isoprostanes; AQP4: Aquaporin-4; d: day; h: hour.

Phenolic Acids	TBI model	Species	Route/ concentration	Regimen	Time of the brain collection	Main results	Reference
Cinnamic acid 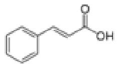	Weight drop	C57Bl/6 mice	Oral gavage/ 100 mg/kg	Daily, 30 consecutive days after TBI	30 days after TBI	↑ neurological function<br>↑ memory<br>↓ brain edema<br>↓ abnormalities in the synapses<br>Restored synaptic spine density<br>Restored synaptophysin protein levels<br>↓ HDAC2 protein levels	[126]
Gallic acid 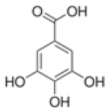	Weight drop	Wistar rats	Oral gavage/ 100 mg/kg	Daily 7 consecutive days before the TBI	8h after TBI	↑ neurological score<br>↑ memory task performance<br>↓IL-1β, IL-6, TNF-α protein levels<br>↓MDA levels<br><br>Improved electrophysiological parameters (PS and fEPSP)	[83]
	Weight drop	Wistar rats	Oral gavage/ 100 mg/kg	Daily, from 7 days before to 2 days after TBI	48h after TBI	↑ memory task performance<br>↓ IL-1β, IL-6, TNF-α protein levels<br><br>Improved electrophysiological parameters (PS, PS AUC and fEPSP)	[84]
Protocatechuic acid 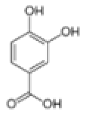	CCI	Sprague-Dawley rats	IP / 30 mg/kg	Daily for 7 consecutive days, starting immediately after TBI	24h and 7 days after TBI	↓ neurodegeneration (24h)<br>↓ oxidative injury (24h)<br>↓ GSH depletion (24h)<br>↓ dendritic loss (24h)<br>↓ microglial activation (7d)<br>↓ delayed neuronal cell death (7d)	[122]
Ferulic acid 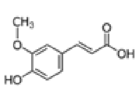	Weight drop	Wistar rats	IP / 100 mg/kg	Single injection immediately after TBI	48h after TBI	↓ neuronal apoptosis<br>↑ SOD, GPx levels<br>↓ MDA levels	[123]

Caffeic acid phenethyl ester 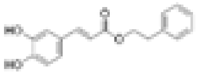	CCI	Sprague-Dawley rats and C57BL/6 mice	IP / 10mg/kg	Single injection, 30 min after TBI for IHC studies or injection 30 min after TBI and daily for 4 consecutive days for behavioural studies	24h after TBI	↓ cortical contusion volume in rats<br>↑ claudin-5 in rats<br>↓ vascular dysfunction in rats and mice<br><br>No improvement of motor or cognitive function in rats	[127]
	Weight drop	Sprague-Dawley rats	IP / 10mg/kg	30 min after TBI, and then repeated daily for 7 days		↓ F2-IsoPs levels in the blood	[124]
	Weight drop	Sprague-Dawley rats	IP / 10mg/kg	30 min after TBI, and then repeated daily for 7 days	24 hand 7 days after the TBI	↓ AQP4 mRNA expression in the brain (4 and 7d)	[128]
	Weight drop	Sprague-Dawley rats	IP / 10 μmol/kg	Single injection 15 min after TBI	4h after TBI	↓ MDA levels<br>↑ SOD and GPx levels<br>↓ degenerative changes in neurons<br>↓ caspase-3 immunoreactivity<br><br>Protection of neuronal morphology	[125]

Table S9. Ellagic acid effects on TBI models after oral and intraperitoneal administration. TBI: traumatic brain injury; IP: intraperitoneal injection; IL-1β: interleukin 1 beta; IL-6: interleukin 6; BBB: blood-brain barrier; PS: Population spike amplitude; fEPSP: Field excitatory postsynaptic potential; TNF-α : tumor necrosis factor alpha; d: day; h: hour.

**Ellagic acid**	**Species**	**Route/ concentration**	**Regimen**	**Time of the brain collection**	**Main results**	**Reference**
Ellagic acid 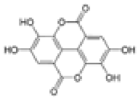	Wistar rats	Oral gavage/ 100 mg/kg	Daily 7 consecutive days before the TBI	48h after TBI	↑ clinical signs<br>↓ learning and memory deficits<br>↓ IL-1β and IL-6 protein levels<br>↑ BBB integrity<br>Restored electrophysiological parameters (PS and fEPSP)	[82]
	Wistar rats	IP / 100 mg/kg	Immediately after trauma and then once	48h after TBI	↑ neurological score<br>↑ cognitive performance	[129]

	every 8hr until 48hr	↓ TNF-α protein levels<br><br>Improved electro-physiological parameters (PS)	
